# Supplementary material for: Therapeutic Performance Evaluation of 213Bi-Labelled Aminopeptidase N (APN/CD13)-Affine NGR-Motif ([213Bi]Bi-DOTAGA-cKNGRE) in Experimental Tumour Model: A Treasured Tailor for Oncology
Source: Pharmaceutics. 2023 Feb 1;15(2):491. doi: 10.3390/pharmaceutics15020491 (PMC9968005; doi:10.3390/pharmaceutics15020491)
Supplement: Supplementary file 1 [file pharmaceutics-15-00491-s001.zip › pharmaceutics-2181096-supplementary.pdf]

Therapeutic Performance Evaluation of ^{213}Bi-labelled Aminopeptidase N (APN/CD13)-affine NGR-motif ([^{213}Bi]Bi-DOTAGA-cKNGRE) in Experimental Tumor Model: A Treasured Tailor for Oncology

Zita Képes[1,*], Viktória Arató[1,2], Judit P. Szabó[1], Barbara Gyuricza[1,3], Dániel Szücs[1,3,4], István Hajdu[1], Anikó Fekete[1], Frank Bruchertseifer[5], Dezső Szikra[1] and György Trencsényi[1]

1 Division of Nuclear Medicine and Translational Imaging, Department of Medical Imaging, Faculty of Medicine, University of Debrecen, Nagyerdei St. 98, H-4032 Debrecen, Hungary
2 Doctoral School of Pharmaceutical Sciences, University of Debrecen, Nagyerdei St. 98, H-4032 Debrecen, Hungary
3 Doctoral School of Chemistry, Faculty of Science and Technology, University of Debrecen, Egyetem square 1, H-4032 Debrecen, Hungary
4 Department of Physical Chemistry, Faculty of Science and Technology, University of Debrecen, Egyetem square 1, H-4032 Debrecen, Hungary
5 European Commission, Joint Research Centre (JRC), Karlsruhe, Germany
* Correspondence: kepes.zita@med.unideb.hu

Table S1. *Ex vivo* biodistribution of [^{213}Bi]Bi-DOTAGA-cKNGRE in HT1080 tumor-bearing mice 30 and 90 minutes postinjection of approximately 5 MBq of the APN/CD13 selective radiotracer. The uptake values were obtained as mean %ID/g±SD. n=8 mice/time point. Significance level between 30 and 90 minutes for the corresponding radiopharmaceutical: $p \leq 0.01$ (**).

Organ	30 min	90 min
**blood**	2.72±0.22	0.38±0.33**
**liver**	0.72±0.35	0.89±0.64
**spleen**	0.99±0.26	0.34±0.20
**kidney**	11.36±1.41	4.51±1.21**
**small intestine**	0.69±0.20	0.16±0.08**
**large intestine**	0.71±0.06	0.26±0.08**
**stomach**	0.73±0.04	0.32±0.28
**muscle**	0.49±0.09	0.06±0.02**
**fat**	0.72±0.05	0.18±0.25
**lung**	2.09±0.74	0.71±0.44
**heart**	0.62±0.18	0.18±0.10
**brain**	0.08±0.05	0.004±0.001
**bone (femur)**	0.69±0.38	0.08±0.05
**salivary gland**	0.79±0.53	0.26±0.31
**gall bladder**	0.49±0.31	0.17±0.24
**pancreas**	0.57±0.18	0.08±0.03**
**HT1080 tumor**	1.48±0.18	0.80±0.16

Table S2. *Ex vivo* tumor-to-organ ratios 30 and 90 minutes postinjection of approximately 5 MBq of the APN/CD13 selective [^{213}Bi]Bi-DOTAGA-cKNGRE. The values were obtained as mean %ID/g of the tumor/mean %ID/g of the organ (±SD). n=8 mice/time point. Significance level between 30 and 90 minutes for the corresponding radiopharmaceutical: $p \leq 0.01$ (**).

Tumor-to-organ	30 min	90 min
**Tumor/blood**	1.83±0.21	0.26±0.05**
**Tumor/liver**	0.49±0.08	0.60±0.12**
**Tumor/spleen**	0.67±0.10	0.23±0.08
**Tumor/kidney**	7.67±1.59	3.04±0.98
**Tumor/small intestine**	0.47±0.09	0.10±0.01**
**Tumor/large intestine**	0.48±0.04	0.17±0.02
**Tumor/stomach**	0.49±0.06	0.22±0.04
**Tumor/muscle**	0.33±0.02	0.04±0.01**
**Tumor/fat**	0.48±0.08	0.12±0.03**
**Tumor/lung**	1.41±0.07	0.48±0.07**
**Tumor/heart**	0.42±0.08	0.12±0.02
**Tumor/brain**	0.05±0.01	0.003±0.001
**Tumor/bone (femur)**	0.46±0.08	0.06±0.01**
**Tumor/salivary gland**	0.53±0.12	0.17±0.03
**Tumor/gall bladder**	0.33±0.06	0.12±0.04
**Tumor/pancreas**	0.39±0.07	0.05±0.01**
